# TwinBLAST: When Two Is Better than One

**DOI:** 10.1128/MRA.00842-19

**Published:** 2019-08-29

**Authors:** Julie C. Dunning Hotopp, James Matsumura, Robin E. Bromley, David R. Riley, Sonia Agrawal, Ben Sparklin, John Mattick, Jonathan Crabtree, Anup Mahurkar

**Affiliations:** a Institute for Genome Sciences, University of Maryland School of Medicine, Baltimore, Maryland, USA; b Department of Microbiology and Immunology, University of Maryland School of Medicine, Baltimore, Maryland, USA; c Greenebaum Cancer Center, University of Maryland School of Medicine, Baltimore, Maryland, USA; Indiana University, Bloomington

## Abstract

Analysis of sequence read pairs can be essential for characterizing structural variation, including junction-spanning pairs of reads (JSPRs) suggesting recent lateral/horizontal gene transfer. TwinBLAST can be used to facilitate this analysis of JSPRs by enabling the visualization and curation of two BLAST reports side by side in a single interface.

## ANNOUNCEMENT

Lateral and horizontal gene transfer (LGT and HGT, respectively) are common in bacteria (1, 2), which exchange DNA to increase variance in the absence of sexual recombination. LGT can even occur between very diverse taxa, such as bacteria and animals. There is a plethora of recent LGTs from *Wolbachia* endosymbionts to their hosts (3–8). Recent LGTs can be identified by the presence of junction-spanning read pairs (JSPRs) between donor and recipient genomes with tools such as LGTSeek (9) with manual examination of the BLAST search results for pairs of sequencing reads. To this end, we developed a flexible tool called TwinBLAST to enable visual inspection and curation of two BLAST (10) reports simultaneously.

TwinBLAST is available to users through either the source code (https://github.com/IGS/twinblast) or a preconfigured virtual machine (VM; https://sourceforge.net/projects/twinblast/files/) that has all the necessary dependencies installed, as well as example data. TwinBLAST is a Web-based utility with the interface implemented in Ext JS JavaScript and the server-side code implemented in Perl, making use of BioPerl (11) modules for BLAST file parsing/indexing, CGI for argument handling, and Bio::Graphics for rendering alignment visuals. A MySQL database is present in the backend to enable curation of the read pairs. The installation and usage of TwinBLAST are outlined in an online tutorial (https://docs.google.com/document/d/1YKzd8pH05Wd5dB5cNLmo_Q6AKyEQbGz4dmiIecjG6Ho/edit?usp=sharing) and YouTube video (https://www.youtube.com/watch?v=FUqoxEIGML0&list=PLT3OVYkIByoHAOIu1ZxV-undAxsUf3cbg&index=3).

**FIG 1:**
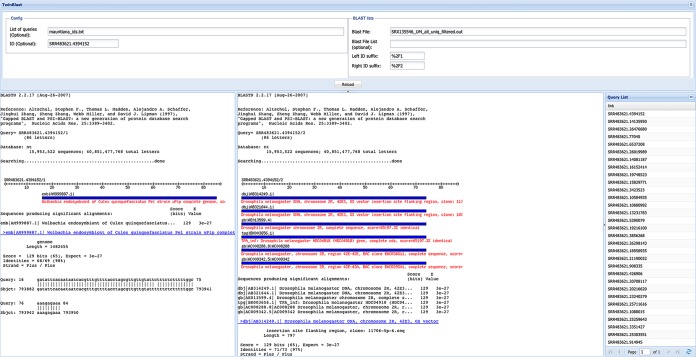
Screen capture of a public instance of Twin-BLAST with putative JSPRs from a *Wolbachia* endosymbiont to *Drosophila mauritiana* identified in strain mau12w (SRA number SRA050824). Optional panels have double arrows in the upper right that facilitate hiding the panels. All panels are shown here, but the top panel is hidden by default.

The TwinBLAST interface (e.g., http://lgt.igs.umaryland.edu/twinblast/) has four panels (Fig. 1). The two largest panels each contain an independently scrollable hyperlinked BLAST report, one for each read in a read pair. Along the entire top is the configuration panel (Fig. 1), which is used for loading the data and is hidden by default. On the right side of the configuration panel are places to specify BLAST output files for both reads and the identification (ID) suffix used to distinguish the two reads. The ID prefix free-form text box allows an ID to be specified, such that the BLAST reports for the ID will be displayed in the corresponding boxes on the left and right sides of the display. There is an option when setting up a private TwinBLAST interface to have radial buttons enabling curation. Lastly, a query list can be provided on the left side of the configuration panel that populates the navigation and curation panel.

TwinBLAST has greatly increased our ability to rapidly validate and curate putative JSPRs, aiding in the identification of putative LGTs. For example, we identified putative JSPRs in public data from Drosophila mauritiana mau12w (SRA number SRA050824) (12), where one read in a pair is initially identified as matching a *Wolbachia* reference genome from Wolbachia pipientis strain *w*Mel (13) and *Wolbachia* sp. strains *w*Ri (14) and *w*Pip (15) (GenBank accession numbers NC_002978, NC_010981, and NC_012416) using BWA ALN version 0.5.9-r16 (16) with default parameters, while the other read in the pair did not map. These read pairs will include (i) putative JSPRs that could indicate *Wolbachia*-host LGT and/or (ii) junctions between a conserved and unique region in the query *Wolbachia* genome. Therefore, subsequently, these read pairs are searched against the NCBI NT database using BLASTN, visualizing and curating the results in TwinBLAST based on both the taxonomy of the BLAST matches for both reads in the pair and the complexity of the sequences. A manually curated subset is provided (http://lgt.igs.umaryland.edu/twinblast/) that includes only read pairs where one read matches a *Wolbachia* sp. and the other read in the pair matches the insect. This curation suggests that further experiments aimed at examining LGT from a *Wolbachia* endosymbiont to this line of *D. mauritiana* are justified and warranted.

### Data availability.

A public instance of TwinBLAST is available for testing at http://lgt.igs.umaryland.edu/twinblast/. The source code is available at https://github.com/IGS/twinblast. The preconfigured virtual machine is available at https://sourceforge.net/projects/twinblast/files/. An online tutorial document is available at https://docs.google.com/document/d/1YKzd8pH05Wd5dB5cNLmo_Q6AKyEQbGz4dmiIecjG6Ho/edit?usp=sharing. A YouTube tutorial is available at https://www.youtube.com/watch?v=FUqoxEIGML0&list=PLT3OVYkIByoHAOIu1ZxV-undAxsUf3cbg&index=3.

## References

[1] Beiko RG, Harlow TJ, Ragan MA 2005 Highways of gene sharing in prokaryotes. Proc Natl Acad Sci U S A 102:14332–14337. doi:10.1073/pnas.0504068102. 16176988 PMC1242295

[2] Puigbo P, Wolf YI, Koonin EV 2010 The tree and net components of prokaryote evolution. Genome Biol Evol 2:745–756. doi:10.1093/gbe/evq062. 20889655 PMC2997564

[3] Hotopp JCD, Clark ME, Oliveira DCSG, Foster JM, Fischer P, Torres MCM, Giebel JD, Kumar N, Ishmael N, Wang S, Ingram J, Nene RV, Shepard J, Tomkins J, Richards S, Spiro DJ, Ghedin E, Slatko BE, Tettelin H, Werren JH 2007 Widespread lateral gene transfer from intracellular bacteria to multicellular eukaryotes. Science 317:1753–1756. doi:10.1126/science.1142490. 17761848

[4] Kondo N, Nikoh N, Ijichi N, Shimada M, Fukatsu T 2002 Genome fragment of *Wolbachia* endosymbiont transferred to X chromosome of host insect. Proc Natl Acad Sci U S A 99:14280–14285. doi:10.1073/pnas.222228199. 12386340 PMC137875

[5] Aikawa T, Anbutsu H, Nikoh N, Kikuchi T, Shibata F, Fukatsu T 2009 Longicorn beetle that vectors pinewood nematode carries many *Wolbachia* genes on an autosome. Proc Biol Sci 276:3791–3798. doi:10.1098/rspb.2009.1022. 19692404 PMC2817283

[6] McNulty SN, Foster JM, Mitreva M, Dunning Hotopp JC, Martin J, Fischer K, Wu B, Davis PJ, Kumar S, Brattig NW, Slatko BE, Weil GJ, Fischer PU 2010 Endosymbiont DNA in endobacteria-free filarial nematodes indicates ancient horizontal genetic transfer. PLoS One 5:e11029. doi:10.1371/journal.pone.0011029. 20543958 PMC2882956

[7] Fenn K, Conlon C, Jones M, Quail MA, Holroyd NE, Parkhill J, Blaxter M 2006 Phylogenetic relationships of the *Wolbachia* of nematodes and arthropods. PLoS Pathog 2:e94. doi:10.1371/journal.ppat.0020094. 17040125 PMC1599763

[8] Desjardins CA, Cerqueira GC, Goldberg JM, Dunning Hotopp JC, Haas BJ, Zucker J, Ribeiro JM, Saif S, Levin JZ, Fan L, Zeng Q, Russ C, Wortman JR, Fink DL, Birren BW, Nutman TB 2013 Genomics of *Loa loa*, a *Wolbachia*-free filarial parasite of humans. Nat Genet 45:495–500. doi:10.1038/ng.2585. 23525074 PMC4238225

[9] Riley DR, Sieber KB, Robinson KM, White JR, Ganesan A, Nourbakhsh S, Dunning Hotopp JC 2013 Bacteria-human somatic cell lateral gene transfer is enriched in cancer samples. PLoS Comput Biol 9:e1003107. doi:10.1371/journal.pcbi.1003107. 23840181 PMC3688693

[10] Altschul SF, Gish W, Miller W, Myers EW, Lipman DJ 1990 Basic local alignment search tool. J Mol Biol 215:403–410. doi:10.1016/S0022-2836(05)80360-2. 2231712

[11] Stajich JE, Block D, Boulez K, Brenner SE, Chervitz SA, Dagdigian C, Fuellen G, Gilbert JG, Korf I, Lapp H, Lehvaslaiho H, Matsalla C, Mungall CJ, Osborne BI, Pocock MR, Schattner P, Senger M, Stein LD, Stupka E, Wilkinson MD, Birney E 2002 The Bioperl toolkit: Perl modules for the life sciences. Genome Res 12:1611–1618. doi:10.1101/gr.361602. 12368254 PMC187536

[12] Garrigan D, Kingan SB, Geneva AJ, Andolfatto P, Clark AG, Thornton KR, Presgraves DC 2012 Genome sequencing reveals complex speciation in the *Drosophila simulans* clade. Genome Res 22:1499–1511. doi:10.1101/gr.130922.111. 22534282 PMC3409263

[13] Wu M, Sun LV, Vamathevan J, Riegler M, Deboy R, Brownlie JC, McGraw EA, Martin W, Esser C, Ahmadinejad N, Wiegand C, Madupu R, Beanan MJ, Brinkac LM, Daugherty SC, Durkin AS, Kolonay JF, Nelson WC, Mohamoud Y, Lee P, Berry K, Young MB, Utterback T, Weidman J, Nierman WC, Paulsen IT, Nelson KE, Tettelin H, O’Neill SL, Eisen JA 2004 Phylogenomics of the reproductive parasite *Wolbachia pipientis w*Mel: a streamlined genome overrun by mobile genetic elements. PLoS Biol 2:e69. doi:10.1371/journal.pbio.0020069. 15024419 PMC368164

[14] Klasson L, Westberg J, Sapountzis P, Naslund K, Lutnaes Y, Darby AC, Veneti Z, Chen L, Braig HR, Garrett R, Bourtzis K, Andersson SG 2009 The mosaic genome structure of the *Wolbachia w*Ri strain infecting *Drosophila simulans*. Proc Natl Acad Sci U S A 106:5725–5730. doi:10.1073/pnas.0810753106. 19307581 PMC2659715

[15] Klasson L, Walker T, Sebaihia M, Sanders MJ, Quail MA, Lord A, Sanders S, Earl J, O'Neill SL, Thomson N, Sinkins SP, Parkhill J 2008 Genome evolution of *Wolbachia* strain *w*Pip from the *Culex pipiens* group. Mol Biol Evol 25:1877–1887. doi:10.1093/molbev/msn133. 18550617 PMC2515876

[16] Li H, Durbin R 2009 Fast and accurate short read alignment with Burrows-Wheeler transform. Bioinformatics 25:1754–1760. doi:10.1093/bioinformatics/btp324. 19451168 PMC2705234

